# Electrophysiological evidence for retrieval mode immediately after a task switch

**DOI:** 10.1016/j.neuroimage.2014.12.068

**Published:** 2015-03

**Authors:** Lisa H. Evans, Angharad N. Williams, Edward L. Wilding

**Affiliations:** Cardiff University Brain Research Imaging Centre (CUBRIC), School of Psychology, Cardiff University, Cardiff CF10 3AT, Wales, UK

**Keywords:** Retrieval mode, Episodic memory, Task-switching, Event-related potentials (ERPs), Task-set, Retrieval preparation

## Abstract

It has been suggested that retrieving episodic information can involve adopting a cognitive state or set: retrieval mode. In a series of studies, an event-related potential (ERP) index of retrieval mode has been identified in designs which cue participants on a trial-by-trial basis to switch between preparing for and then completing an episodic or non-episodic retrieval task. However, a confound in these studies is that along with task type the content of what is to be retrieved has varied. Here we examined whether the ERP index of retrieval mode remains when the contents of an episodic and non-episodic task are highly similar – both requiring a location judgement. In the episodic task participants indicated the screen location where words had been shown in a prior study phase (left/right/new); whereas in the perceptual task they indicated the current screen location of the word (top/middle/bottom). Consistent with previous studies the ERPs elicited while participants prepared for episodic retrieval were more positive-going at right-frontal sites than when they prepared for the perceptual task. This index was observed, however, on the first trial after participants had switched tasks, rather than on the second trial, as has been observed previously. Potential reasons for this are discussed, including the critical manipulation of similarity in contents between tasks, as well as the use of a predictable cue sequence.

## Highlights

• People were cued to switch between episodic and non-episodic cognitive tasks.
• Both tasks required judgements about stimulus location.
• ERPs were acquired in response to the cues signalling which task to complete.
• Preparatory ERPs for episodic retrieval had different timings than in prior studies.
• These outcomes offer new insights into processes that facilitate episodic retrieval.

## Introduction

Episodic memory retrieval involves a number of processes. Most frequently researchers focus on the downstream consequences of the interaction between a retrieval cue and a memory representation. Another important factor, however, is the cognitive state of the individual before a retrieval cue is encountered or generated. Tulving (1983) argued that in order for a person to remember a particular episode they need to enter a cognitive set where stimulus items are treated as retrieval cues – known as retrieval mode. Neuroimaging methods have been useful in studying retrieval mode due to the difficulty in assessing cognitive states using behavioural methods alone. Retrieval mode is thought to be a sustained cognitive set that is entered when there is a requirement to retrieve episodic information and, consequently, can be revealed by contrasting neural activity while people are preparing to complete different kinds of task e.g. episodic versus non-episodic (Rugg and Wilding, 2000).

A series of positron emission tomography (PET) and functional magnetic resonance (fMRI) studies have indicated that the right prefrontal cortex is involved in the initiation and/or maintenance of retrieval mode (Lepage et al., 2000; Nyberg et al., 1995; Velanova et al., 2003). This is also consistent with the findings in a study by Düzel et al. (1999, 2001) who recorded direct current (DC) potentials while participants switched between completing separate blocks of an episodic task (old/new recognition judgement) and a semantic task (animacy judgement). Each block had four words and a cue was presented prior to the first word to indicate which task participants should complete. Electrical activity was more positive-going at a right frontopolar site during episodic than semantic retrieval. This difference emerged around the time that the task cue was presented, increased until the second word and was then maintained for the rest of the block. Extended analysis by Düzel et al. (2001) found that the DC potential differences observed between the episodic and semantic tasks could be modelled by a generator in the right prefrontal cortex. They interpreted this difference as an index of retrieval mode.

Further event-related potential (ERP) studies have been conducted using designs where activity has been contrasted on a trial-by-trial basis to determine with increased specificity the dynamics of retrieval mode. Morcom and Rugg (2002) used this type of design and the same tasks as Düzel et al. (1999, 2001). Neural activity was time-locked to the cue denoting which task participants should prepare to complete before test words appeared 1.6 s later. They found that neural activity elicited by the episodic cue was relatively more positive-going than the semantic cue at right fronto-central scalp locations, from approximately 500 ms post-cue until the onset of the test word. This effect was evident on the second successive trial after a task switch (hereafter a ‘stay’ trial) but not on the first trial of a task (a ‘switch’ trial). Morcom and Rugg (2002) suggested that the adoption of retrieval mode takes time and may not be completed until at least one item has been presented which requires a response.

Since these studies a further three similar ERP studies have been conducted. Herron and Wilding (2004) asked the participants to switch between three different tasks: semantic (a moving/non-moving judgement) or one of two episodic tasks (recover spatial information or encoding task from the study phase). Both of the episodic preparatory cues elicited a greater relative positivity compared to the semantic cue over right-frontal electrode locations from around 800 ms until the onset of the test word (2300 ms after the preparatory cue). These findings further corroborate the interpretation of this effect as a neural index of processes linked with retrieval mode due to it being present across two different episodic tasks relative to the semantic task and invariant between the episodic tasks. Consistent with the findings of Morcom and Rugg (2002), Herron and Wilding (2004) also found that this effect was present on stay trials only. In a further paper (Herron and Wilding, 2006a), in which the preparatory period was extended to 4000 ms, they again found that ERPs following episodic and semantic cues diverged on stay trials at right-frontal scalp locations. This suggests that time to prepare is not the only determinant in the adoption of retrieval mode. Wilckens et al. (2011) also found divergences between an episodic cue (old/new recognition) and a semantic cue (animacy task) on stay trials only. However, in contrast to all previous studies it was the semantic cue which generated more positive-going ERPs than the episodic cue. This discrepancy might be due to a different reference electrode being used or the use of pictures in this study compared to words in all other studies.

A key observation for the work reported here is that in order to assess retrieval mode researchers have manipulated the tasks participants complete, but these tasks have also differed in terms of the content that is to be retrieved subsequently. In the studies by Düzel et al. (1999, 2001), Morcom and Rugg (2002) and Wilckens et al. (2011) participants made an old/new recognition judgement (episodic task) and an animate/inanimate decision (semantic task). The tasks used by Herron and Wilding (2004, 2006a) required participants to make a left/right/new judgement (episodic), an animacy/pleasantness/new decision (episodic; 2004 paper only) or a moving/not moving/unsure choice (semantic). Therefore it is unclear whether the neural differences that have been observed between episodic and semantic cues reflects retrieval mode, the consensual interpretation, or the differences between the contents of what is to be retrieved.

The aim of this study was to assess the possibility that differences between contents are responsible for the divergences in preparatory activity described above, rather than reflecting preparation for episodic retrieval per se. This was accomplished by manipulating retrieval mode by having only one of the two tasks requiring episodic retrieval, as has been done previously, but crucially keeping the contents of the tasks very similar. To achieve this, one task entailed the recovery of location information from a study phase (episodic task) and required a location judgement (left/right/new). The other task also involved a location judgement but this time of the current screen location of the test word (perceptual task) and a top/middle/bottom decision was required. These tasks both entailed a location judgement that was similar, rather than identical, to minimise conflict between the response options given that they were usually incongruent between the tasks. If the activity that has been observed previously, the right-frontal greater relative positivity elicited by episodic cues in comparison to non-episodic cues, is an index of retrieval mode, then it should also be evident in this study. The absence of this effect would challenge current accounts of the processes engaged during preparation for episodic retrieval.

## Method

### Participants

Forty-eight individuals participated in the study for payment of £15 after giving informed consent. All were right-handed native English speakers aged 18–30 and 35 were females. Sixteen participants were excluded from the experiment: 11 failed to contribute sufficient artefact free trials in the conditions of interest (≥ 16; see Evans et al., in press, for related test item data), and 5 fell below the threshold for behavioural performance (defined as < .6 source discrimination, see below). Thus 32 participants (24 females) were included in the study.

### Design

Stimuli consisted of 240 concrete nouns selected from the MRC psycholinguistic database (Coltheart, 1981) with Kucera–Francis frequencies of 1–9 per million. All words had between 3 and 9 letters and were presented in Times New Roman font in white letters on a black background. The words were randomly assigned to one of 20 lists each containing 12 words. There were 10 study–test cycles. Within each cycle, one list was shown at study and again at test along with a second list. No lists were repeated across cycles. Half of the study words were presented on the left half of the monitor and half on the right. The designation of words to the left or right side of the screen was counterbalanced across participants and presented in a randomised order.

During the test phase words were shown slightly above, at, or below fixation, with an equal number at each location. Each of these words was preceded by one of two preparatory cues, indicating which task participants were to complete, and these were denoted by the capital letters O and X. The mapping of these letters to task was counterbalanced across participants. Each test cue type was always presented for two consecutive trials. The test stimuli were presented on a monitor 1.2 metres from the participant and subtended a maximum visual angle of 2.1° vertically and 2.5° horizontally. The old/new status of words and the assignment of words to the episodic or perceptual task were fully counterbalanced.

### Procedure

Each study–test block started with a message on screen indicating the number of the block participants were about to complete. At study participants were asked to indicate whether the word appeared on the right or left side of the screen. A fixation asterisk was presented for 1000 ms, then a word for 300 ms. The monitor was then blanked until a response was made, after which the monitor remained blank for a further 500 ms before the start of the next study trial. Participants responded with their index and middle fingers, counterbalanced across left and right hand. Left side location judgements were always associated with the leftmost of the two fingers.

At test each trial started with a preparatory cue indicating which task participants should prepare to complete. One cue indicated that participants should prepare to decide whether the word was new (not shown at study) or had appeared on the left or right side of the screen – the episodic task. The other cue directed participants to prepare to indicate whether the test word had just appeared toward the top, middle or bottom of the monitor – the perceptual task. Each task required one of three responses; episodic task: left/right/new and perceptual task: top/middle/bottom. The preparatory cue stayed on screen for 300 ms, followed by a fixation asterisk for 2000 ms, then the test word for 300 ms. The monitor was then blanked until participants made a response, and remained blank for a further 500 ms before the next preparatory cue was shown. Participants were asked to pay attention to the preparatory cue in order to identify the impending task requirements and to respond accordingly. Participants responded using the same fingers as at study, with the addition of the index finger of the other hand to indicate new or below fixation. They were encouraged to balance speed and accuracy equally.

### Electroencephalogram (EEG) acquisition

EEG was recorded during the test phase from 25 silver/silver chloride electrodes embedded in an elastic cap, and from two electrodes placed on the left and right mastoids. The recording locations were based on the International 10–20 system (Jasper, 1958) and included midline (Fz, Cz, Pz), fronto-polar (Fp1/Fp2), frontal (F7/F8, F5/F6, F3/F4), central (T7/T8, C5/C6, C3/C4), posterior (P7/P8, P5/P6, P3/P4) and occipital (O1/O2) sites. Vertical and horizontal eye movements were recorded from additional electrodes placed above and below the left eye (VEOG) and from the outer canthi (HEOG). EEG was recorded at 167 Hz and then down-sampled offline to 84 Hz and referenced to linked mastoids. EEG and EOG were recorded with a bandwidth of 0.03–40 Hz (− 3 dB). Trials containing large EOG artefact were rejected, as were trials containing A/D saturation or baseline drift exceeding ± 80 μV. Other EOG blink artefacts were corrected using a linear regression estimate (Semlitsch et al., 1986). A 7-point binomially weighted smoothing filter was applied prior to analysis.

## Results

The term episodic hit will be used for correct location judgments to old words in the episodic task and perceptual hit for accurate responses to the location of words in the perceptual task. Correctly classified new test items in the episodic task are referred to as correct rejections. Trials where the test cue was the same as on the preceding trial are referred to as stay trials, while trials where the test cue was different from the prior trial are called switch trials. A significance level of p < .05 was adopted for all analyses.

### Behavioural data

In the study phase the likelihood of a correct left or right judgement was at ceiling. Table 1 shows the accuracy and associated reaction time (RT) data separated according to whether responses were made on switch or stay trials. For the episodic task, the likelihood of a correct old response to an old word, irrespective of the accuracy of location judgments, was greater than the likelihood of an old response to a new word for switch and for stay trials [means of 0.93 vs 0.09 in both cases: t(31)s > 39.52, p < .001]. For both switch and stay trials the mean accuracy of location judgments was reliably above chance [both t(31)s > 13.49, p < .001].

An ANOVA was conducted on the accuracy data (hits) with factors of task (episodic, perceptual) and trial type (switch, stay). There was a task by trial type interaction, [F(1,31) = 4.43, p < .05] and a main effect of task, [F(1,31) = 96.87, p < .001] but no main effect of trial type. The interaction reflected a small but nonetheless reliable improvement in accuracy in the perceptual task across switch and stay trials [t(31) = 2.27, p < .05] that was not evident in the episodic task.

A parallel ANOVA was also conducted on the RT data. There was a main effect of task, [F(1,31) = 121.96, p < .001] and a main effect of trial type, [F(1,31) = 12.88, p = .001] but no interaction involving these factors. A RT switch cost was evident in the episodic [t(31) = 2.28, p < .05] and perceptual task [t(31) = 3.18, p < .01].

### ERP analyses

Mean amplitudes were calculated time-locked to the preparatory task cues and separated according to the trial type. The mean numbers of trials (ranges in parentheses) contributing to each condition of interest were as follows: episodic switch = 53 (41–60), episodic stay = 54 (36–60), perceptual switch = 54 (40–60) and perceptual stay = 54 (43–60).

**Fig. 1:**
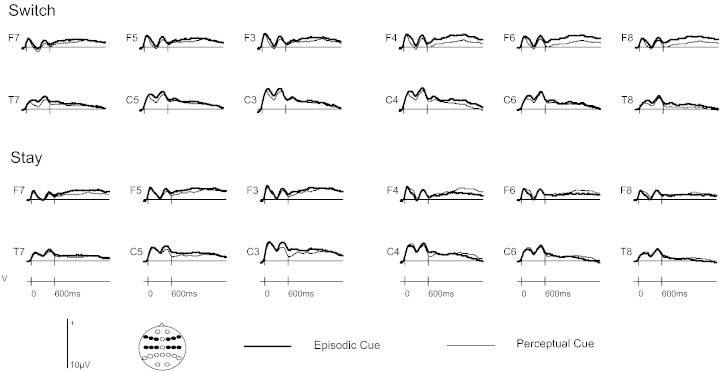
Grand average preparatory cue-related ERPs separated according to cue type (episodic/perceptual) and trial type (switch/stay).

In previous ERP studies the putative index of retrieval mode has been evident from around 800 ms until the end of the preparatory period (Herron and Wilding, 2004, 2006a; Morcom and Rugg, 2002; Wilckens et al., 2011). In this study the time-window of 800–1900 ms was used, following Herron and Wilding (2004). Furthermore, the electrophysiological correlate of retrieval mode is prominent at frontal and fronto-central sites and is right-lateralised (Düzel et al., 1999, 2001; Herron and Wilding, 2004, 2006a; Morcom and Rugg, 2002). Therefore in this study the initial analysis of the ERPs included 12 sites distributed over fronto-central regions (F3/F4, F5/F6, F7/F8, C3/C4, C5/C6, T7/T8), again following Herron and Wilding (2004). To gain an overview of the electrophysiological data see Fig. 1.

An ANOVA on the preparatory cue activity was conducted with factors of cue type (episodic, perceptual), trial type (switch stay), location in the anterior–posterior dimension (frontal, central), hemisphere (left, right) and site (inferior, mid-lateral, superior). Only outcomes involving cue or trial type factors are reported. There was a significant interaction involving cue type, trial type, anterior–posterior dimension and hemisphere [F(1,31) = 6.99, p < .05] as well as a lower-order interaction involving cue type, trial type and hemisphere [F(1,31) = 12.80, p = .001].

Consistent with previous studies (e.g. Herron and Wilding, 2004, 2006a) follow-up analyses were carried out separately for switch and stay trials. For switch trials there was a cue type x anterior–posterior axis x hemisphere interaction [F(1, 31) = 4.44, p < .05] and a lower-order cue type by anterior–posterior interaction [F(1,31) = 5.78, p < .05]. There were no significant results involving cue type at central sites. By contrast, examining anterior sites revealed a cue type x hemisphere interaction [F(1,31) = 7.85, p < .01] and a main effect of cue type [F(1,31) = 4.12, p < .05]. This interaction arose because of a difference between episodic and perceptual cues at right hemisphere frontal locations [F(1,31) = 9.54, p < .01] but not left hemisphere frontal sites. These analyses demonstrate that the episodic cue was more positive-going than the perceptual cue at right frontal electrode sites, consistent with the waveforms presented in Fig. 1.

On the stay trials there was an interaction between cue type and hemisphere, F(1,31) = 8.55, p < .01. Following this up by examining the difference between the episodic and semantic cues in each hemisphere separately revealed no significant main effects of cue type. It is likely that this interaction arose due to a small reversal in polarity between the two cue types across hemispheres.

**Fig. 2:**
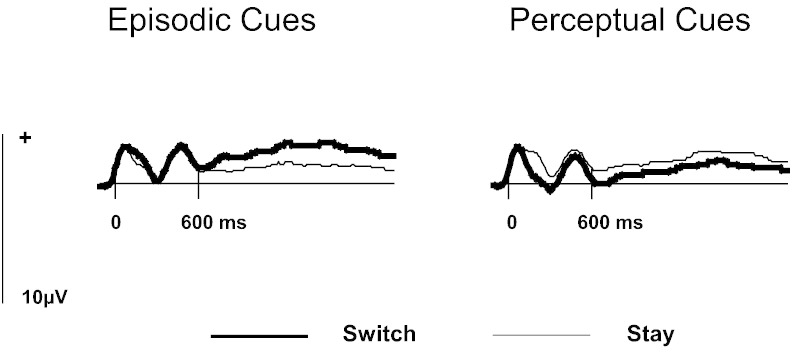
Grand average ERPs elicited on switch and stay trials by the episodic and perceptual cues at the average of right frontal sites (F4, F6, F8) where the index of retrieval mode is of greatest magnitude.

In order to determine if the differences observed between switch and stay trials were due to changes associated with the episodic or the perceptual task we conducted further analyses on each of these tasks separately. These followed the same analysis structure as above, including the factors of: trial type (switch, stay), location in the anterior–posterior dimension (frontal, central), hemisphere (left, right) and site (inferior, mid-lateral, superior). For the perceptual task there were no significant effects involving trial type (p > .1). By contrast, for the episodic task there was an interaction between trial type, the anterior–posterior dimension, hemisphere and site [F(1.8,55.4) = 3.44, p < .05] and two lower-order interactions involving some of the same factors: trial type x anterior–posterior x hemisphere [F(1,31) = 6.73, p < .05] and trial type and hemisphere [F(1,31) = 26.13, p < .001]. Focusing on right frontal sites indicated a relatively greater positivity on switch than on stay trials [F(1,31) = 4.05, p = .05]. Fig. 2 displays grand average waveforms for the average of the three right frontal sites for the episodic and perceptual tasks separately.

## Discussion

The aim of this study was to determine if the electrophysiological differences that have been observed when individuals are preparing to retrieve episodic versus non-episodic information reflect retrieval mode, as has usually been supposed, or are related to differences between the contents participants are preparing to retrieve. In this study this potential confound in the interpretation of previous studies was ameliorated by making the judgement in both tasks about stimulus location. Reliable differences were found between the two cue types from 800 to 1900 ms post-cue, with more positive-going activity following the episodic than the perceptual cue at right frontal scalp locations. This time frame and scalp distribution is entirely in line with previous research on preparatory retrieval processing (Herron and Wilding, 2004, 2006a; Morcom and Rugg, 2002). It is possible to conclude, therefore, that the right-frontal modulation seen in previous studies, and in this one, is consistent with it being an index of retrieval mode: it does not appear to reflect content differences across episodic and non-episodic tasks.

The novel finding in this study is that this neural index of retrieval mode was observed on switch trials only. In previous work it has been evident on stay trials (Herron and Wilding, 2004, 2006a; Morcom and Rugg, 2002; Wilckens et al., 2011), and it has thus been proposed that completing one complete trial in a task is necessary for retrieval mode to be adopted fully (Morcom and Rugg, 2002). The results of this study, however, indicate that this is not true under all circumstances. The most obvious difference between this study and previous ones is the similarity in content between the two tasks here. At first consideration it is possible that similarity in content might interfere with or facilitate the transition between tasks. However, examining the broader task switching literature it has been found that behavioural switch costs are commonly smaller when changing between similar tasks compared to switches between dissimilar tasks (Arrington et al., 2003). One way in which these authors defined similarity was in terms of the similarity of perceptual encoding operations e.g. a form judgement (height and width) and a surface property judgement (hue and brightness). The switch cost was greater between these two types of judgements than within. The authors argued that this is because similar tasks share task-set components which makes it easier to switch between them. In the current study, because location judgements are required in the two tasks, the degree of cognitive reconfiguration required when switching between them is likely to be smaller than in previous studies, which could result in the index of retrieval mode being observed on the earlier switch trial. A similar argument has been made when individuals switch between two episodic tasks, where cue related activity has been found to diverge on the switch trial of the sequence (Herron and Wilding, 2006b; Johnson and Rugg, 2006). It has been suggested that this is because of the greater degree in overlap of cognitive operations between two episodic tasks compared to an episodic and semantic task (Herron and Wilding, 2006a). Therefore, when switching between tasks where less configuration is required, perhaps due to similarity in cognitive operations or content, it is possible that resources can be mobilised more easily.

Another potentially important factor which might determine the time period over which retrieval mode can be adopted is the predictability of the task sequence. In most of the key ERP studies described in the Introduction an unpredictable sequence was used (Herron and Wilding, 2006a; Morcom and Rugg, 2002; Wilckens et al., 2011). The exception to this is Herron and Wilding (2004). There were the same number of trials of each type before a switch, however, in their study participants switched between three different tasks, and the order of the cues was randomised in each block. This would have rendered the task sequence unpredictable to the participant because after completion of one task they would not know which of the other two tasks they would complete next. The outcome of the current study raises the possibility that the use of a predictable task sequence in this experiment contributed to task-specific preparation being observed on switch trials. It might be that a predictable trial sequence encourages the more rapid and complete adoption of a task-set. This assertion also fits in with behavioural research in the task switching domain: when the sequence is predictable the switch cost is limited to the first trial but when it is unpredictable there is a more gradual return to asymptotic performance (Monsell et al., 2003).

In this study participants were quicker to respond on stay than on switch trials in both tasks. There was a small accuracy benefit in the perceptual task, but this was not the case in the episodic task, either in recognition or source memory. The lack of a switch accuracy benefit in the episodic task has been reported previously (Morcom and Rugg, 2002; Wilckens et al., 2011, Experiment 1). However this might have been due to the requirement to only make an old/new recognition judgement. According to dual-process models of memory this judgement can be made on the basis of two independent processes: familiarity, which is a scalar strength signal, and recollection, which involves recovery of qualitative (contextual) information about a prior encounter (Mandler, 1980; Yonelinas, 2002). Morcom and Rugg (2002) suggested that the adoption of retrieval mode might be linked to the availability of recollection to make a memory judgement. Therefore if the episodic task does not require the retrieval of contextual information effects of accuracy across serial position are less likely to be observed because familiarity can also be used to make recognition judgements. Subsequent studies have utilised a source memory task in these switching paradigms, but the results have been mixed. Herron and Wilding (2004), in a design where participants switched between three tasks (two episodic and one semantic), found no accuracy benefit across trial type. Wilckens et al. (2011; Experiment 2) using the same design did, however, find an improvement. Finally, in the later paper by Herron and Wilding (2006a), with one episodic and one semantic task, there were benefits in accuracy which extended across switch, stay and stay + 1 trials. One commonality between the latter two experiments is that the interval between the cue and the test stimulus was 4000 ms, whereas in Herron and Wilding (2004) and the current study this interval was shorter: 2000 ms.

These data are relevant to the question of what the functional significance of retrieval mode is. Tulving (1983) initially stated that “The same stimulus reminds a person of a particular episode only when the individual's mind is in a particular state; the episodic system must be in the ‘retrieval mode’ before a stimulus change in the environment can serve as an effective retrieval cue to store episodic information” (p.46). It is difficult to test the idea that adopting a retrieval mode is a necessary precursor for episodic retrieval, and it is also worth noting that this does not seem to be an intuitively obvious component of a system where rapid access to salient information is desirable in order to guide behaviour. This observation does not preclude the possibility, of course, that retrieval mode might facilitate the recovery of episodic memories. In all of the studies conducted in this area thus far there were RT benefits across the trial sequence (Düzel et al., 1999; Herron and Wilding, 2004; 2006a; Morcom and Rugg, 2002; Wilckens et al., 2011). Thus the adoption of retrieval mode seems to be linked to either greater ease in recovering mnemonic information or subjecting it to task-relevant processing. In this study, where retrieval mode was adopted on the switch trial, it might be anticipated that RTs would be quicker on this trial compared to other studies where it has been found on the later stay trial. Unfortunately it is not possible to get an indication of whether this hypothesis might be valid due to other differences between this study and previous ones: such as in the encoding task, the cue to stimulus interval, and the type and difficulty of the non-episodic task. Although it seems clear that retrieval mode facilitates the time-course of retrieval processing the particular way(s) in which it might do this remains largely unspecified. Some suggestions have been made with respect to constraining the retrieval search space (Buckner, 2003), whereas others argue that preparation maximises the efficiency of search operations or the efficacy with which retrieved information is processed (Herron and Wilding, 2004).

A further question concerns the specific relationship between the neural index observed on switch trials and the process of retrieval mode. One possibility is that this activity, as well as that in other studies where a comparable index has been observed, reflects processes responsible for the adoption of retrieval mode. These may not be entirely the same as those involved in maintaining mode (for a similar argument, see Herron and Wilding, 2006b). The way in which ERPs were employed here is not well-suited for addressing this question, because brain activity that is invariant over trials cannot be detected, hence the appropriate contrast cannot be made. Progress on this question will be made via further work with DC potentials (cf. Düzel et al., 1999), and with fMRI designs which allow a separation between transient and sustained patterns of neural activity (e.g. Donaldson et al., 2001).

In the current experiment participants were quicker and more accurate in performing the non-episodic task compared to the episodic task. Therefore there is a confound between these tasks in terms of their level of difficulty. On the basis of this study alone it might be possible to argue that the right-frontal ERP effect observed on the switch trials might reflect something like the initial perceived difficulty when switching to episodic retrieval. This interpretation, however, seems very unlikely when other studies in the area are also considered. Probably the strongest evidence against this interpretation comes from the study by Herron and Wilding (2004) who contrasted ERPs evoked by cues while participants switched between two episodic tasks and a semantic task. There were no significant differences in accuracy across the three tasks and overall the reaction times for the semantic task lay between those for the two episodic tasks. A common right-frontally distributed positive modulation separated preparation for both kinds of episodic retrieval from preparation for semantic retrieval. Furthermore, Herron and Wilding (2006a) conducted a further cue study where participants switched between an episodic and a semantic task. There was no significant difference in accuracy between the tasks and reaction times were marginally longer in the semantic task. The episodic cues were more positive-going than the semantic cues at right-frontal scalp locations. These studies demonstrate that the presence of the right-frontal modulation cannot be explained by difficulty, as indexed by accuracy or reaction times.

In this experiment, as well as manipulating the similarity of the content between the episodic and non-episodic task, the type of non-episodic task was also different to what has traditionally been used. In all previous ERP studies of retrieval mode the non-episodic task has been a semantic memory task (Düzel et al., 1999, 2001; Herron and Wilding, 2004, 2006a; Morcom and Rugg, 2002; Wilckens et al., 2011). In this study the non-episodic task was a perceptual one. Retrieval mode should be evident regardless of the identity of the baseline task – as long as it does not require episodic retrieval. However, when assessing the presence of retrieval mode the non-episodic task acts as the baseline; therefore it is important that this remains relatively stable between switch and stay trials and any differences which arise do so because of the episodic task. Critically, there were reliable differences between switch and stay trials in the episodic task at right frontal scalp locations, but these were not found in the perceptual task (see Fig. 2). Therefore in this study changes between switch and stay trials stemmed from differences in activity in the episodic task and not the perceptual task. This extends the generality of results from previous studies by demonstrating that the neural index of retrieval mode is also present when a non-semantic task is used.

In summary, the present study demonstrates that when individuals switch between an episodic and a non-episodic task, where the content information is very similar, there is an electrophysiological difference between these preparatory activities at right frontal scalp locations. This outcome suggests that this index does not reflect differences in content between tasks. The novel finding here is that this index was seen on the switch trial of the sequence, rather than the second successive trial, as has been observed previously. It is possible that this outcome arose because the similarity in content required less cognitive configuration when switching between tasks than was the case in previous studies.

## Figures and Tables

**Table 1:** Response accuracy and associated RTs for the episodic and perceptual tasks on switch and stay trials (standard deviations in parentheses).

	Switch	Stay
*Response accuracy*		
Episodic hits	0.79 (0.11)	0.79 (0.12)
Correct rejections	0.91 (0.10)	0.91 (0.09)
Perceptual hits	0.95 (0.06)	0.97 (0.04)
*RTs (ms)*		
Episodic hits	1523 (437)	1459 (379)
Correct rejections	1323 (375)	1235 (275)
Perceptual hits	880 (182)	826 (170)
